# Recurrent laryngeal never monitoring versus non-monitoring in parathyroid surgery

**DOI:** 10.3389/fendo.2023.1299943

**Published:** 2023-11-28

**Authors:** Yongliang Mu, Xuehai Bian, Junjie Yang, Yang Li, Yushuai Zhang, Gianlorenzo Dionigi, Yishen Zhao, Hui Sun

**Affiliations:** ^1^ Department of Thyroid Surgery, China-Japan Union Hospital of Jilin University, Jilin Provincial Key Laboratory of Translational Medicine in Surgery, Jilin Provincial Engineering, Laboratory of Thyroid Disease Prevention and Treatment Changchun, Changchun, China; ^2^ Division of Surgery, Istituto Auxologico Italiano IRCCS (Istituto di Ricovero e Cura a Carattere Scientifico), Milan, Italy; ^3^ Department of Pathophysiology and Transplantation, University of Milan, Milan, Italy

**Keywords:** primary hyperparathyroidism, secondary hyperparathyroidism, parathyroidectomy, intraoperative neural monitoring, IONM, recurrent laryngeal nerve, morbidity, pain

## Abstract

**Background:**

Although intraoperative neural monitoring (IONM) is well established in thyroid surgery, it is less commonly analyzed in parathyroid operations. This study presents the results of IONM for primary and secondary hyperparathyroidism surgery.

**Methods:**

We retrospectively assessed 270 patients with primary hyperparathyroidism (PHPT), 53 patients with secondary hyperparathyroidism (SHPT), and 300 patients with thyroid cancer from June 2010 to June 2022 in one hospital in China. The follow-up was 12 months. Demographic, electromyography data from IONM, laboratory, and clinical information were collected. Laryngoscopy was collected from 109 patients with PHPT in whom IONM was not used. All groups were assessed by Pearson’s chi-square test and Fisher’s exact probability method to verify the relationship between parathyroid size and location, duration of surgery, preoperative concordant localization, laryngeal pain, IONM outcomes, cure rate, and RLN injury. Visual analog scale (VAS) assessed laryngeal pain. RLN outcomes were measured according to nerves at risk (NAR).

**Results:**

The study comprehended 918 NAR, that is 272, 105, 109, and 432 NAR for PHPT, SHPT with IONM, PHPT without IONM, and thyroid surgery control group, respectively. IONM successfully prevented RLN injury (P<0.001, P=0.012): Fifteen (5.51%) RLNs experienced altered nerve EMG profiles during surgery, and five (1.84%) experienced transient RLN injury in PHPT patients. Five (4.76%) RLNs were found to have altered EMG profiles during surgery, and one (0.95%) RLN had a transient RLN injury in SHPT patients. There was no permanent nerve injury (0.00%) in this series. There was no association between location, gland size, preoperative concordant localization, cure rate, duration of surgery, and IONM (P >0.05). Duration of surgery was associated with postoperative pharyngeal discomfort (P=0.026, P=0.024). Transient RLN injury was significantly lower in patients with PHPT who underwent IONM than in those who did not. Intraoperative neuromonitoring played an effective role in protecting the recurrent laryngeal nerve (P=0.035). Compared with parathyroidectomy, thyroidectomy had a higher rate of RLN injury (5.32%, P<0.001).

**Conclusion:**

IONM for SHPT and PHPT offers rapid anatomical gland identification and RLN functional results for effective RLN protection and reduced RLN damage rates.

## 1 Introduction

Parathyroidectomy (PTX) is an effective treatment for drug-refractory primary hyperparathyroidism (PHPT) and secondary hyperparathyroidism (SHPT) (1, 2). PHPT and SHPT have a high prevalence in China, and the incidence of PHPT and SHPT is increasing every year (3, 4).

Recurrent laryngeal nerve (RLN) injuries occur during PTX (5–11). The prevalence of transient RLN injury is 0.8% to 10.6% (8–11). The probability of permanent RLN injury ranges from 0.0% to 14.0% (8–11).

Although intraoperative nerve monitoring (IONM) is well-established in thyroid surgery, it is less frequently analyzed in parathyroid surgery (12–14). According to the international IONM guidelines, nerve monitoring is recommended in parathyroid surgery, but this suggestion is based on evidence from thyroid surgery, not parathyroid surgery (15).

This study presents the results of IONM in primary and secondary hyperparathyroidism surgery, focusing on surgical success rate, RLN outcomes, size and location of the parathyroid, duration of surgery, and pain compared to patients undergoing surgery without IONM.

## 2 Materials and methods

### 2.1 Time frame, patients, and setting

From June 2010 to June 2022, 270 patients with PHPT, 53 SHPT, 300 patients with thyroid cancer treated intraoperatively with IONM, and 109 patients with PHPT who did not receive IONM from the Department of Thyroid Surgery, China-Japan Union Hospital, Jilin University, China.

### 2.2 Ethics

Study registration number: 20230630016. The study was approved by the Institutional Review Board. Patients or their legal guardians will sign a detailed informed consent form before surgery.

### 2.3 PHPT and SHPT epidemiology in China

PHPT patients are increasing year by year. The number of patients with asymptomatic hyperparathyroidism is on the rise and has exceeded 50 percent of all patients until now (3). The number of patients with SHPT is increasing year by year, and according to statistics, the prevalence of CKD in China is estimated at 10.8 percent (4).

### 2.4 Inclusion and exclusion criteria

#### 2.4.1 Inclusion criteria

We analyzed patients operated on for PHPT and SHPT compared with patients operated on for thyroid cancer with IONM and patients operated on for PHPT without IONM. Only patients with pre- (L1) and post-laryngoscopy (L2) were included on the first postoperative day. Patients with or without imaging concordant were included. All patients enrolled were ≥ 18 years old. Reoperation patients and patients with preoperative RLN injury included.

#### 2.4.2 Exclusion criteria

(i) Patients with incomplete data or incomplete follow-up. (ii) Patients with concomitant thyroidectomy. (iii) Patients without L1 and/or L2. (iiii) Tertiary hyperparathyroidism and multiple endocrine neoplasms were excluded.

### 2.5 Definitions

PHPT was diagnosed by the presence of hypercalcemia and a concomitant elevated or inappropriately normal serum PTH level - specifically, PTH > 20 pg/ml with a serum Ca level of > 2.6 mmol/L. The diagnosis of asymptomatic PHPT (aPHPT) was based on the absence of typical symptoms or signs associated with hypercalcemia; the diagnosis was made incidentally on serum Ca level testing or neck ultrasound (US). SHPT is the release of increased amounts of parathyroid hormone, which is an appropriate response to a low calcium or vitamin D level to try to restore calcium levels to normal. Recurrent SHPT is defined as PTH <300 pg/ml within 6 months after surgery and PTH >300 pg/ml after 6 months again. Persistent SHPT is defined as PTH consistently >300 pg/ml after surgery. Laboratory tests included serum Ca (reference range, 2.00-2.60 mmol/L), serum phosphate (0.60-1.60 mmol/L), fasting blood glucose (FBS; 3.7-6.0 mmol/L), alkaline phosphatase (AKP; 50-135 U/L), serum creatinine (sCr; 58-133 umol/L), PTH (15-65 pg/mL) and 25-hydroxyvitamin D (25(OH)D; ≥30 ng/mL). Patients were referred and followed up by their referring endocrinologist.

### 2.6 Indications for surgery in SHPT patients

Patients with SHPT who undergo surgical treatment are mainly those who have failed medical treatment, whose PTH cannot be controlled within 9 times the normal reference value, and who have complications caused by HPT, or those who are inclined to undergo surgical treatment. The major complications are (1) uncontrolled hypercalcemia or hyperphosphatemia; (2) calcification defense or systemic severe extraosseous calcification; (3) cortical bone fracture; (4) weakened limb muscles and bone or joint pain that affects the quality of life; (5) uncontrolled itching that leads to lesions and/or affects the quality of life; and (6) patients with proximity to renal transplantation at risk of severe post-transplant hypercalcemia (16).

### 2.7 Preoperative localization examinations

Preoperative localization was performed by cervical ultrasound (US) and 99mTc-labelled sestamibi image. If the localization scans matched, the abnormal gland was identified using a focused minimally invasive approach, and if the scans did not match, standard bilateral neck exploration was performed.

### 2.8 Intraoperative gland localization

The localization (originating from the right superior, right inferior, left superior, or left inferior parathyroid gland) was determined perioperatively. The superior and inferior parathyroid glands may be very close to each other on a craniocaudal axis, but the typical landmark that was always used was the position of the parathyroid glands and any adenoma relative to the RLN. The upper parathyroid glands typically lie dorsal to the plane of the RLN, while the lower parathyroid glands lie ventral to the plane of the RLN, and in adenomatous enlargement, the migratory pathways of the upper and lower gland adenomas tend to respect this plane.

### 2.9 Intraoperative PTH

IOPTH values were initially checked after induction of anesthesia. They were then rechecked 30 minutes after surgical removal of the parathyroid gland. A > 50% drop in IOPTH levels 30 minutes after removal was used to confirm the successful removal of the abnormal gland.

### 2.10 Treatment

Surgical procedures were divided into (a) conventional: bilateral cervical exploration, visualization of all parathyroid glands, and removal of the pathological gland(s). (b) focused by open minimally invasive approach: identification and targeted removal of the gland(s) identified as pathological.

### 2.11 Variables

Sex, age, BMI, preoperative serum calcium, postoperative serum calcium, preoperative serum phosphorus, preoperative parathyroid hormone (PTH), IOPTH, alkaline phosphatase (ALP), IONM data, laryngoscopy data, parathyroid gland location and size, course of RLN, postoperative paraffin pathology. The most common postoperative complications were recorded (transient or permanent hypocalcemia; unilateral or bilateral paralysis of the vocal cords).

### 2.12 Grouping method

We analyzed: (a) the effect of IONM on RLN injury in PHPT and SHPT patients with IONM. (b) IONM compared to PHPT patients without IONM. (c) RLN injury rate compared to patients operated on for thyroid cancer in the same study period.

### 2.13 IONM technique

The RLN was monitored (Nerve Integrity Monitor 3.0 from Medtronic (USA)) according to the “four-step RLN monitoring method” proposed by Chiang et al. (17), i.e., V1, R1, V2, and R2 were recorded. During the surgery, intermittent monitoring was the main method, we continuously monitored the patient’s RLN with the IONM when the nerves were dissected. In the event of RLN injuries, we have archived all information, i.e. possible cause of injury, anatomical details of the parathyroid, and anatomical details of the nerve. The operating steps of the IONM are shown in **Table 1**. V1 and V2 were stimulated without dissection of the carotid sheath.

**Table 1:** Operational steps for IONM.

Operation	Note
IONM Four-Step Approach	
Step 1, V1 signal	A significant EMG signal was obtained by probing the ipsilateral vagus nerve at the level of the inferior pole of the thyroid gland (point B) (proving the successful establishment of the monitoring system). If there is no signal at point B, the vagus nerve is probed at the level of the upper pole of the thyroid gland (point A), and a signal is obtained at point A, confirming the presence of a non-returning laryngeal nerve variant.
Step 2, R1 signal	Before revealing the RLN, use a probe to “cross” the trachea perpendicularly and then parallel to the trachea in the area of its course. Positioning of the RLN and monitoring of the EMG signals after exposure to the RLN
Step 3, R2 signal	Continuous monitoring during dissection of the RLN and real-time comparison of signal changes. After full exposure, the EMG signals are obtained at the nearest end of the exposure.
Step 4, V2 signal	Detection of vagal EMG signals after complete hemostasis in the operative field and before closing the incision.
Signal Interpretation	
R2, V2 signal not significantly attenuated	Functional integrity of the RLN
R2, V2 signal loss	Damage to the RLN during a surgical operation, and explore the nerve’s “damage point” to find the cause of the damage

### 2.14 RLN follow-up

Intraoperative temporary nerve signal abnormalities were defined as the loss of nerve signal intraoperatively but recovery of nerve signal at the end of surgery. Intraoperative persistent nerve signal abnormalities are defined as the loss of nerve signal intraoperatively until the end of surgery. Patients with abnormal vocal fold movements were reexamined by laryngoscopy at the second, fourth, and sixth postoperative weeks.

Transient RLN injury is defined as symptoms of neuropraxia resolving within six months. Permanent RLN injury is defined as symptoms of neuropraxia persisting for six months.

### 2.15 Assessment of postoperative pharyngeal pain

The visual analog scale (VAS) (18) was used to assess the degree of pharyngeal pain experienced by patients on the first day after surgery.

### 2.16 Pathology

Paraffin pathology was done on all parathyroid glands removed and all pathology reports were recorded in detail.

### 2.17 Statistical methods

Statistics are for nerves at risk(NAR), not patients.SPSS 27.0 software was used for statistical analysis. Measurement data were expressed as mean ± standard deviation; count data were expressed as frequency and percentage (%). The Kruskal-Wallis rank sum test was used to compare continuous variables, the Pearson chi-square test was used for categorical variables, and Fisher’s exact probability method was used to compare theoretical frequencies < 1. P < 0.05 was statistically significant, and P < 0.01 was statistically significant.

## 3 Results

### 3.1 Basic information

#### 3.1.1 Basic population

**Figure 1:**
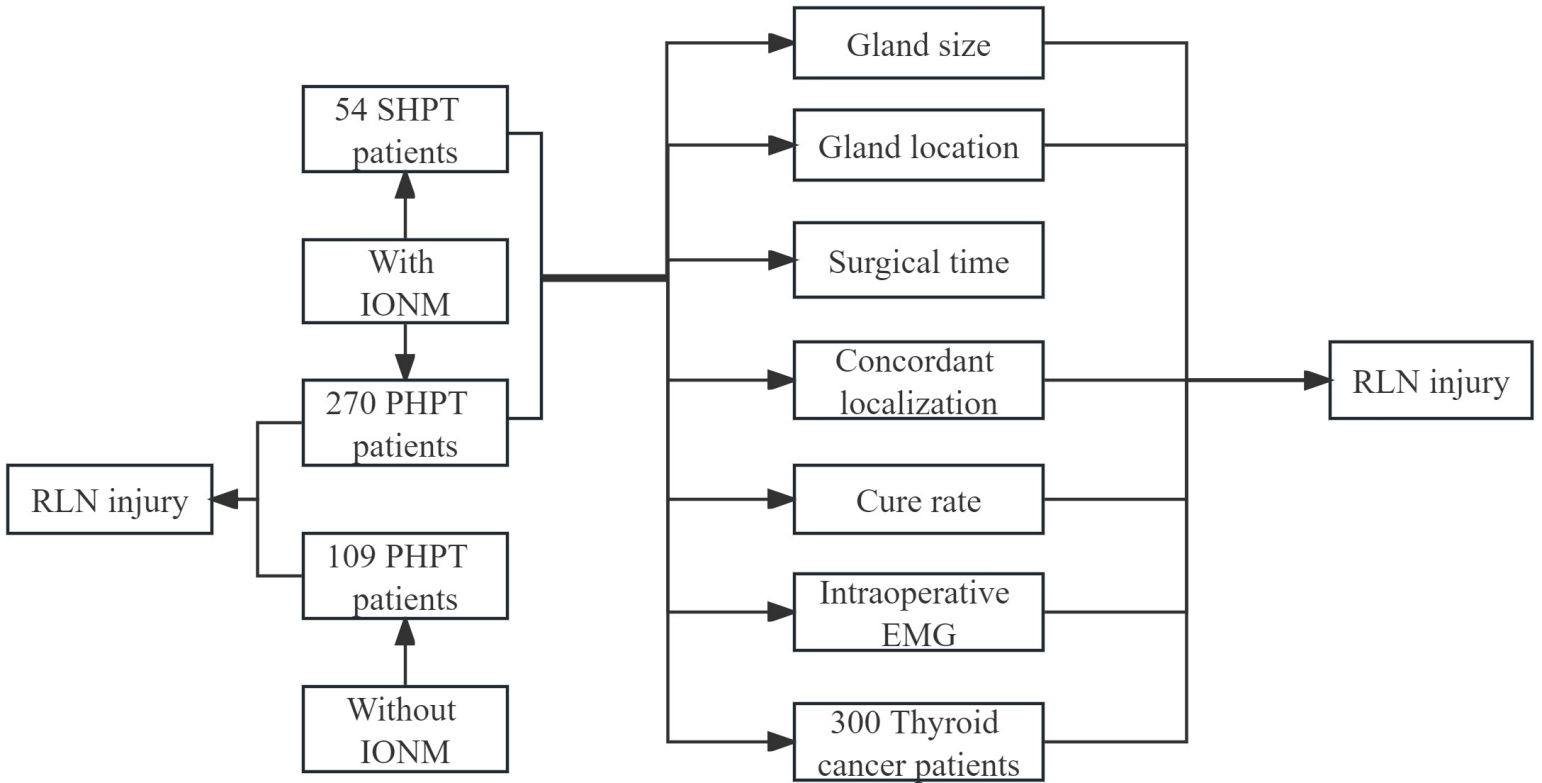
Flowchart of different groups for RLN injury analyzing.

The study consists of 270 patients with PHPT, 53 SHPT, 300 patients with thyroid cancer treated intraoperatively with IONM, and 109 patients with PHPT who did not receive IONM. Some patients did not use IONM because of financial problems or for their own reasons. See **Figure 1**.

A higher percentage of female patients (74.4%) than male patients (25.6%) participated in the study with PHPT. The ratio of male to female patients at SHPT was 2.5:1. PHPT and SHPT patients were statistically significantly different in terms of age, preoperative serum calcium, preoperative serum phosphorus, preoperative alkaline phosphatase, preoperative PTH, intraoperative PTH, calcitonin, parathyroid length and duration of surgery (P < 0.05). See **Tables 2**, **3**.

**Table 2:** Distribution of the number of different groups of patients with basic information.

Variables	Grouping	Number	Total
PHPT patients			
Gender	Male	69 (25.66%)	270
	Female	201 (74.44%)	
Parathyroid location	Superior parathyroid	63 (22.02%)	285
	Inferior parathyroid	222 (77.89%)	
Parathyroid size	≥2cm	79 (27.72%)	285
	<2cm	206 (72.28%)	
Parathyroid pathology	adenoma hyperplasia cyst	275(96.49%) 8(2.81%) 2(0.70%)	285
Duration of surgery	≥1.5h	107 (36.63%)	270
	<1.5h	163 (60.37%)	
RLN nerve signals	Normal signal	257 (94.49%)	272
	Temporary nerve signal abnormalities	10 (3.68%)	
	Persistent nerve signal abnormalities	5 (1.84%)	
Follow-up results	Number of cures	269(99.63%)	270
	Number of recurrences	1(0.37%)	
SHPT patients			
Gender	Male	38 (71.70%)	53
	Female	15 (28.30%)	
Parathyroid location	Superior parathyroid	106 (50.00%)	212
	Inferior parathyroid	99 (46.70%)	
	Ectopic parathyroid	7 (3.30%)	
Parathyroid size	≥2cm	71 (33.49%)	212
	<2cm	141 (66.51%)	
Parathyroid pathology	adenoma hyperplasia	200(94.34%) 12(5.66%)	212
Duration of surgery	≥1.5h	43 (81.23%)	53
	<1.5h	10 (18.87%)	
RLN nerve signals	Normal signal	100 (95.24%)	105
	Temporary nerve signal abnormalities	4 (3.81%)	
	Persistent nerve signal abnormalities	1 (0.95%)	
Follow-up results	Number of cures	49(92.45)	53
	Number of persistent	1(1.89%)	
	Number of recurrences	3(5.66%)	

**Table 3:** Statistical values of basic patient information and laboratory indicators.

Variables	Statistical values(Mean ± SD)		*P*
	PHPT patients	SHPT patients	
Age(years)	51.59 ± 10.70	46.17 ± 9.732	<0.001
BMI	24.06 ± 7.24	23.02 ± 3.13	0.299
Preoperative serum calcium(mmol/L)	2.90 ± 0.32	2.54 ± 0.20	<0.001
Postoperative serum calcium(mmol/L)	2.29 ± 0.23	2.21 ± 0.35	0.063
Preoperative serum phosphorus(mmol/L)	0.89 ± 0.24	2.50 ± 0.55	<0.001
Preoperative alkaline phosphatase(mmol/L)	214.27 ± 322.77	562.77 ± 511.35	<0.001
Preoperative PTH(pg/mL)	515.57 ± 616.51	2153.10 ± 888.50	<0.001
Intraoperative PTH(pg/mL)	49.72 ± 68.75	176.50 ± 85.05	<0.001
PTH decline rate	0.88 ± 0.09	0.91 ± 0.04	0.226
Calcitonin(pg/mL)	1.10 ± 2.59	12.83 ± 13.90	<0.001
Parathyroid length(cm)	1.86 ± 0.33	1.51 ± 0.51	<0.001
Duration of surgery(mins)	89.4 ± 23.90	107.30 ± 17.62	<0.001

#### 3.1.2 Pathology

In the PHPT group, more diseased parathyroid glands were located in the lower thyroid (77.89%) and less than 2 cm (72.28%); surgery lasted less than 1.5 hours in most patients (60.37%). In the SHPT group, 7 ectopic parathyroid glands were found, of which, 2 parathyroid glands were in the carotid sheath and 5 parathyroid glands were in the thymic horn. See **Table 2**.

Of 285 parathyroid glands in PHPT patients, adenomas were seen in 275 (96.49%), hyperplasia in eight (2.81%), and cysts in two (0.70%). Of 212 parathyroid glands of SHPT patients, adenomas appeared in 200 (96.34%) and hyperplasia in 12 (5.66%). See **Table 2**.

#### 3.1.3 Follow-up examination

In this study, patients were followed up for 1 year after surgery. We found that: after 1 year, the cure rate of PHPT patients was 99.63% and one patient (0.37%) had recurrence. The cure rate of SHPT was 92.45%, three (5.66%) patients had recurrence and one (1.89%) patient had persistent hyperparathyroidism. See **Table 2**.

#### 3.1.4 NAR

In the PHPT group, two hundred and seventy-two RLNs were dissected during the procedure. Fifteen RLNs (5.56%) had abnormal RLN signals during the procedure, and five RLNs (1.85%) had abnormal RLN signals at the end of surgery.

In the SHPT group, one hundred and five RLNs were dissected intraoperatively. Five RLNs (9.43%) had abnormal intraoperative RLN signals, and one RLN (1.89%) had abnormal RLN signals at the end of surgery. See **Table 2**.

#### 3.1.5 Laryngeal examination results

In the preoperative laryngoscopy, we found that three patients who underwent reoperation had their right vocal folds fixed in a paramedian position and were hoarse, including two patients with PHPT and one with SHPT; the other patients had good vocal fold movement and no RLN injury. Laryngoscopy was repeated on the first day after surgery. Five (1.85%, 5/270) PHPT patients had transient RLN injury and one (1.89%, 1/53) SHPT patient had transient RLN injury. Laryngoscopy revealed that the left vocal cord was fixed in the paramedian position in four patients and the right vocal fold was fixed in the paramedian position in two patients. Laryngoscopies were performed every fortnight after surgery in six patients, and six weeks after surgery the movement of the bilateral vocal folds was symmetrical and well closed, without hoarseness or cough.

#### 3.1.6 Surgical procedures

In the 270 PHPT patients with intraoperative use of IONM, 2 patients underwent parathyroidectomy/bilateral exploration and the others underwent unilateral exploration. All PHPT patients without intraoperative IONM underwent parathyroidectomy/unilateral exploration. All SHPT patients (53) underwent total parathyroidectomy (bilateral exploration) combined with bilateral central group lymph node dissection. 300 thyroid cancer patients underwent thyroid lobectomy combined with lymph node dissection. The details are shown in **Table 4**.

**Table 4:** Specifics of different surgical procedures.

	Parathyroidectomy/ unilateral exploration	Parathyroidectomy/ bilateral exploration	Total	Number of nerves dissected
PHPT patients with IONM	268	2	270	272
PHPT patients without IONM	109	0	109	109
*SHPT patients with IONM	0	53	53	106
	Thyroid lobectomy combined with lymph node dissection	Total thyroidectomy combined with lymph node dissection		
Thyroid cancer patients with IONM	300	0	300	300

*Surgical scope for SHPT patients includes total parathyroidectomy (bilateral exploration) combined with bilateral central group lymph node dissection.

### 3.2 Relationship between RLN injury and preoperative concordant localization studies

Of the 285 parathyroids in patients with PHPT, a total of 262 (91.93%) parathyroids were localized consistently with the preoperative examination, of which four parathyroids were resected with persistent RLN nerve signal abnormality. Twenty-three (8.07%) parathyroids were localized inconsistently or without clear preoperative localization, and one parathyroid was resected with persistent RLN nerve signal abnormality. Of the 212 parathyroids in patients with SHPT, a total of 173 (81.60%) parathyroids were localized consistently with the preoperative examination, and one parathyroid was resected with persistent RLN nerve signal abnormality. Thirty-nine (18.40%) parathyroids were localized inconsistently or were not localized definitively preoperatively. Statistical findings: under IONM, there was no relationship between preoperative localization of parathyroids and RLN injury in PHPT patients (P=0.396) and SHPT patients (P=0.523). See **Table 5**.

**Table 5:** Table of the relationship between parathyroid Concordant Localization and RLN injury.

	Number of examples	Persistent RLN signal abnormalities	Normal signal	χ^2^	*P*
Parathyroid of PHPT patients					
Concordant localization	262(91.93%)	4 (1.53%)	258 (98.47%)	0.720	0.396
Discordant or negative localization	23(8.07%)	1 (4.35%)	22 (95.65%)		
Total	285	5 (1.75%)	280 (98.25%)		
Parathyroid of SHPT patients					
Concordant localization	173(81.60%)	1 (0.58%)	172 (99.42%)	0.408	0.523
Discordant or negative localization	39(18.40%)	0 (0%)	39 (100%)		
Total	212	1 (0.47%)	211 (99.53%)		

### 3.3 Relationship between glandular location and RLN injury

**Figure 2:**
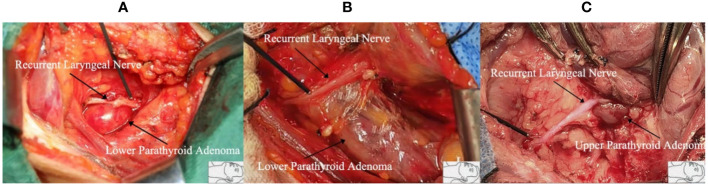
The different positions of the recurrent laryngeal nerve and parathyroid gland. **(A)** Inferior parathyroid adenoma protrudes upward. The recurrent laryngeal nerve is pushed up, and its course becomes shallow and easy to injure; **(B)** Inferior parathyroid adenoma is larger in size, grows toward the middle of the thyroid gland, and has a longer length of concomitant course with the recurrent laryngeal nerve, which makes the dissection of the recurrent laryngeal nerve more difficult; **(C)** Superior parathyroid adenoma is located at the point where the recurrent laryngeal nerve enters the larynx, where the surrounding tissues are dense. It is easy to injure and cause injury when the recurrent laryngeal nerve is separated from the mass.

A total of 285 diseased parathyroid glands were resected in PHPT patients and 212 parathyroid glands in SHPT patients. In IONM, two RLNs had persistent RLN signaling abnormalities after resection of the upper parathyroid gland and three RLNs had persistent RLN signaling abnormalities after resection of the lower parathyroid gland in PHPT patients. One RLN had persistent RLN signaling abnormalities after upper parathyroid resection in SHPT patients. Statistically, there was no association between resection of a differently located parathyroid gland in PHPT (P=0.668) and SHPT patients (P=0.499) and RLN injury with IONM. See **Table 6**. Intraoperative pictures of our center which showed parathyroid glands in different locations with RLN. **Figure 2**.

**Table 6:** Relationship between parathyroid location and RLN injury.

	Number of examples	Persistent RLN signal abnormalities	Normal signal	χ^2^	*P*
PHPT patients					
Superior parathyroid	63	2 (3.17%)	61 (96.83%)	0.184	0.668
Inferior parathyroid	222	3 (1.35%)	219 (98.65%)		
Total	285	5 (1.75%)	280 (98.25%)		
SHPT patients					
Superior parathyroid	106	1 (0.94%)	105 (99.06%)	1.391	0.499
Inferior parathyroid	99	0 (0%)	99 (100%)		
Ectopic parathyroid	7	0	7 (100%)		
Total	212	1 (0.47%)	211 (99.53%)		

### 3.4 Gland size and RLN injury

The mean diameter of the diseased parathyroid gland in PHPT patients was 1.86 cm; seventy-nine parathyroid glands were ≥2 cm long, of which three (3.80%) RLNs had persistent RLN signaling abnormalities. Two hundred and six parathyroid glands were < 2 cm long, and two (0.97%) RLNs had persistent RLN signaling abnormalities. The mean diameter of the diseased parathyroid gland in SHPT patients was 1.51 cm, with 71 parathyroid glands ≥2 cm in length, of which one (1.40%) RLN had persistent RLN signaling abnormalities, and 141 parathyroid glands < 2 cm in length, without RLN injury. In IONM, RLN injury was not associated with parathyroid size in PHPT (P=0.261) and SHPT patients (P=0.138). See **Table 7**.

**Table 7:** The relationship between parathyroid size and RLN injury.

	Number of examples	Persistent RLN signal abnormalities	Normal signal	χ^2^	*P*
PHPT patients					
diameter ≥ 2cm	79	3 (3.80%)	76 (96.20%)	1.261	0.261
diameter <2cm	206	2 (0.97%)	204 (99.03%)		
Total	285	5 (1.75%)	280 (98.25%)		
SHPT patients					
diameter ≥ 2cm	71	1 (1.40%)	70 (98.59%)	2.197	0.138
diameter <2cm	141	0 (0.00%)	141 (100%)		
Total	212	1 (0.47%)	211 (99.53%)		

### 3.5 Cure rate and RLN injury

Of the 270 PHPT patients, all five (1.86%) patients who developed transient RLN damage were from the 269 cured patients. One (2.04%) of the 53 SHPT patients who developed transient RLN damage were from the cured patients. Statistical findings: under IONM, there was no relationship between cure rate, incidence of persistent hyperparathyroidism, recurrence rate, and RLN injury in PHPT patients (P=0.931) and SHPT patients (P=0.924). See **Table 8**.

**Table 8:** The relationship between cure rate and RLN injury.

	Number of examples	Persistent RLN signal abnormalities	Normal signal	χ^2^	*P*
PHPT patients					
Number of cures	269	5 (1.86%)	264 (98.14%)	0.008	0.931
Number of recurrences	1	0 (0.00%)	204 (100%)		
Total	270	5 (1.85%)	269 (99.63%)		
SHPT patients					
Number of cures	49	1 (2.04%)	48 (97.96%)	0.159	0.924
Number of persistent	1	0 (0.00%)	1 (100%)		
Number of recurrences	3	0 (0.00%)	3(100%)		
Total	53	1 (1.89%)	52 (98.11%)		

### 3.6 Duration of surgery and RLN injury and pharyngeal symptoms

**Figure 3:**
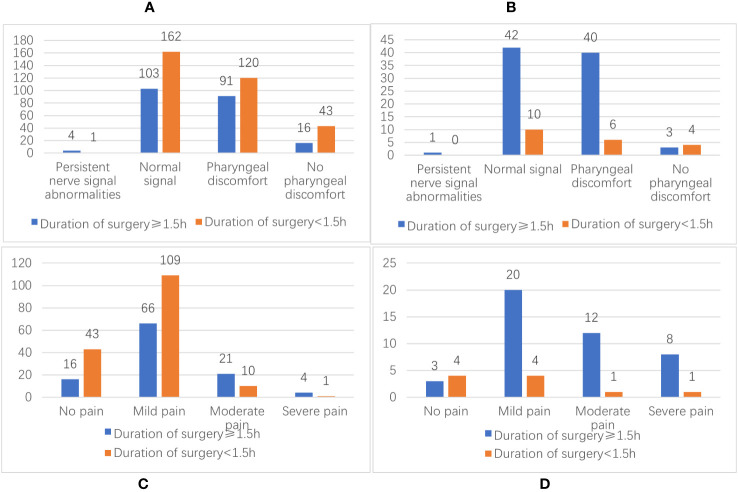
**(A)** Distribution of the duration of surgery and the number of RLN injuries and pharyngeal discomfort in PHPT patients; **(B)** Distribution of the duration of surgery and the number of RLN injuries and pharyngeal discomfort in SHPT patients; **(C)** Distribution of the number of PHPT patients with postoperative pharyngeal discomfort; **(D)** Distribution of the number of SHPT patients with postoperative pharyngeal discomfort.

Of the 107 patients with PHPT whose surgery duration was more than 1.5 hours, four (3.73%) patients had temporary RLN injuries, and 91 (85.05%) patients had pharyngeal discomfort. Of the 163 patients with surgery duration of less than 1.5 hours, one (0.61%) patient was observed with transient RLN injury and 120 (73.62%) patients with pharyngeal discomfort. Forty-three patients with SHPT had a surgery duration of more than 1.5 hours, one (2.32%) patient with a transient RLN injury and 40 (90.67%) patients with pharyngeal complaints were observed. In the ten patients with surgery duration of less than 1.5 hours, there was no RLN injury and six patients (60.00%) had pharyngeal discomfort. The statistical results showed that in IONM, the duration of surgery (χ2 = 1.964, P=0.161; χ2 = 0.423, P=0.516) was not the cause of RLN injury and was a factor in postoperative pharyngeal discomfort (χ2 = 4.939, P=0.026; χ2 = 5.106, P=0.024) (see **Figures 3A, B**. The duration of surgery has a direct correlation with the incidence of postoperative pharyngeal discomfort, with longer surgeries resulting in a higher likelihood of patients experiencing this discomfort. Additionally, those who do experience postoperative pharyngeal discomfort are more likely to report moderate to severe pain. **Figures 3C, D**.

#### 3.6.1 IONM for RLN injury (1)

With IONM, 15 (5.51%) RLNs had abnormal RLN signals, and only five (1.84%) had abnormal RLN signals at the end of surgery in 270 patients with PHPT. In 53 patients with SHPT, five RLNs (4.76%) had abnormal RLN signals during surgery, and only one RLN (0.95%) had abnormal RLN signals at the end of surgery. The statistical results showed that with IONM, the surgeon recognized the possible contributing factors for intraoperative nerve injury in time and avoided the risk. RLN injury was effectively prevented in PHPT patients (P < 0.001) and SHPT patients (P=0.012). See **Table 9**.

**Table 9:** Distribution of the number of RLNs with intraoperative signal abnormalities. .

	Persistent RLN signal abnormalities	Normal signal	Total number of RLNs	χ^2^	*P*
PHPT patients					
Transient RLN signal abnormalities	5 (33.33%)	10 (66.77%)	15	30.776	<0.001
Normal signal	0 (0.00%)	257 (100.00%)	257		
Total	5 (1.84%)	265 (97.43%)	272		
SHPT patients					
Transient RLN signal abnormalities	1 (20.00%)	4 (80.00%)	5	6.294	0.012
Normal signal	0 (0.00%)	100 (100.00%)	100		
Total	1 (0.95%)	104 (99.05%)	105		

#### 3.6.2 IONM and RLN injury (2)

To investigate whether IONM can protect RLN, 270 PHPT patients who used intraoperative IONM and 109 PHPT patients who did not use intraoperative IONM were counted. The rates of temporary RLN injury were 1.84% (5/272) and 5.50% (6/109) for both. The rates of permanent RLN injury were 0.00% and 0.92% (1/109), indicating that using IONM during surgery effectively protected against RLN (P =0.013). See **Table 10**.

**Table 10:** RLN injury under different conditions.

Grouping	Normal RLN	RLN temporary injury	RLN permanent injury	Total number of RLNs	χ^2^	*P*
Intraoperative PHPT patient with IONM	267 (98.16%)	5(1.84%)	0 (0.00%)	272	6.148	0.013
Intraoperative PHPT patient without IONM	102 (93.58%)	6 (5.50%)	1(0.92%)	109		
Patients with hyperparathyroidism	371 (98.41%)	6 (1.59%)	0 (0.00%)	377	13.539	<0.001
Patients with Thyroid cancer	403 (93.29%)	23(5.32%)	6 (1.39%)	432		

### 3.7 Comparison of RLN injury between parathyroidectomy and thyroidectomy

Of the 323 patients who underwent parathyroidectomy with IONM, six (1.59%) RLN had transient RLN and no permanent injury. In comparison, twenty-three (5.32%) RLNs had a transient RLN injury and six (1.39%) RLNs had a permanent RLN injury in 432 RLNs in 300 patients who underwent thyroid lobectomy combined with lymph node dissection. The results showed that the likelihood of RLN injury was higher in patients who underwent radical thyroidectomy (P<0.001). See **Table 10**.

## 4 Discussion

IONM allows rapid localization of the nerve. Patients are characterized by morphological changes in the parathyroid gland, variable anatomical location, variations of the laryngeal nerve, and other features (19, 20). The incidence of the non-recurrent laryngeal nerve is statistically 0.26% to 0.99% on the right side and about 0.04% on the left side (21–24). 60.8% of the RLN runs within the tracheoesophageal groove, 4.9% of the RLN is more lateral to the trachea, and 28.3% of the RLN is directly posterior to the thyroid gland (25). The right RLN is more oblique in the paratracheal region than the left RLN (26); the RLN may be localized with the inferior thyroid artery in as many as 20 different ways (27, 28). Two non-recurrent laryngeal nerves have been identified at our center. Sometimes the arteriovenous thickness and morphology resemble the nerve, which is difficult to see with the naked eye; patients must undergo reoperation if the anatomy has changed, fibrous tissue is proliferating and there are severe tissue adhesions (5). All these conditions affect the accurate localization of the RLN, which in turn compromises the safety of the procedure. By using IONM to strictly implement the “four-step RLN monitoring method”,” continuous intraoperative monitoring, and other technical nerve monitoring procedures (17, 29), the nerve can be quickly localized and differentiated, improving the protection of the nerve and shortening the localization time.

### 4.1 Analysis of the relationship between IONM and preoperative localization of parathyroid glands

Currently, ultrasonography (US), computed tomography (CT), and single-photon emission computed tomography (SPECT)-CT with sestamibi Tc99m can achieve 100% accuracy in the preoperative localization of parathyroid glands (2, 30). Therefore intraoperative exploration of parathyroid glands that are not localized is common. According to the data in this study, there was no relationship between the preoperative accuracy of parathyroid gland localization and RLN injury in PHPT patients and SHPT patients under IONM. This suggests that IONM also plays a role in laryngeal recurrent nerve protection in the exploration and removal of parathyroid glands that fail to be accurately localized. The study by Karakas E et al. (31) also confirms that IONM in combination with other techniques can be effective in protecting the RLN during PHPT surgery.

#### 4.1.1 Analysis of the relationship between IONM and parathyroid gland location

The literature reports that the upper parathyroid gland is mainly located within 2 cm of the junction of the inferior thyroid artery and the RLN in the cricothyroid joint area (4), which is a risk area for RLN injury during upper parathyroid resection. The lower parathyroid gland is mainly located in the lower pole of the thyroid gland, usually on the lateral side of the RLN. Some ectopic parathyroid glands even continue into the anterior mediastinum; in some SHPT patients, a partial thymectomy is required for surgery (32). The above area has a long RLN pathway and is difficult to detect, and it is also a vulnerable area for RLN injury in PTX. In our study, there was no statistically significant effect of parathyroid location on RLN injury, suggesting that the neurological safety of PTX in PHPT and SHPT patients was significantly improved by the use of IONM. The study by Brian R et al. (33) is identical to the present study.

### 4.2 Analysis of the relationship between IONM and parathyroid gland size

Nowadays, more and more surgeons choose to perform resection of the diseased parathyroid gland with a monopolar or bipolar electrometer (34), which has the advantages of less bleeding, faster clotting, and a gentle procedure, but also increases the risk of mild nerve damage such as heat conduction. According to a study by Zhao et al. (35), the distance between the electric knife and the nerve is unsafe within 3 mm. In the present study, it was found that some normal-sized or hyperplastic parathyroid glands may have a smaller distance to the recurrent laryngeal nerve, up to 1.5 mm. In the present study, there was no statistically significant effect of parathyroid gland size on RLN injury, suggesting that the neurological safety of PTX is significantly improved with IONM in PHPT and SHPT patients. One study shared the results of the present study, in which the right upper parathyroid mass was up to 0.25 ± 0.39 cm closer to the RLN compared to other parathyroid glands, and there was no permanent RLN injury after surgery in 136 patients with IONM (33).

IONM enables analysis of the mechanisms of nerve injury, prevention of nerve injury, and appropriate protection of nerves. With IONM, fifteen (5.51%) RLNs and five (4.76%) RLNs had abnormal intraoperative nerve signals in PHPT patients and SHPT patients in this study, and only five (1.84%) RLNs and one (0.95%) RLN, respectively, had abnormal nerve signals at the end of surgery. This is due to the continuous monitoring by the IONM, which provides real-time nerve signals to the surgeon. Any factors affecting the RLN signal are displayed and the surgeon can detect and avoid them in time through the IONM, which really protects the RLN. In an SHPT patient with a 2.1 x 0.9 cm left parathyroid gland, the RLN electromyographic signal R decreased from 1,226 V to 135 V when the parathyroid gland was exposed by pulling the thyroid gland during resection of the left upper parathyroid gland. The signal was restored by exposing the thyroid gland, indicating that the ligaments Berry was stuck in the left RLN, and the RLN signal was normal at the end of surgery. Three hundred and seventy-seven RLNs were identified in this group and dissected using IONM. The rate of transient injury in this group was 1.59%, with no permanent injury, and the rate of RLN injury was much lower than in PHPT patients without IONM (5.50%). It is also much lower than previous reports (6, 9, 36), which fully demonstrates the function of IONM in revealing and analyzing the mechanism of RLN injury and protecting the nerves.

Compared to parathyroidectomy, thyroid surgery poses a greater challenge to the protection of the RLN. In addition to the RLN anatomical factors described above, the infiltration of nerves by thyroid cancer, the greater length of the naked RLN, the presence of perioperative complications, the management of Berry’s ligament, the high likelihood of bleeding and difficulty of hemostasis near the RLN, lymph node dissection of the central group and multiple surgeries may affect the RLN signal (37–39). Therefore, thyroid surgery has a higher probability of RLN injury than parathyroid surgery, with the same statistical results as in the present study, with a probability of transient RLN injury of 5.32% and 1.59%, respectively. The rate of RLN injury in patients with thyroid cancer after IONM currently ranges from 2.6% to 6.0% (9, 14, 40).

Several studies have shown that IONM reduces the time to intraoperative detection of RLN by the surgeon (41, 42). In this study, we found that the duration of the procedure was not related to RLN injury and was associated with patients’ postoperative pharyngeal discomfort. The duration of surgery has a direct correlation with the incidence of postoperative pharyngeal discomfort, with longer surgeries resulting in a higher likelihood of patients experiencing this discomfort. Additionally, those who do experience postoperative pharyngeal discomfort are more likely to report moderate to severe pain. Therefore, IONM might play a role in patients’ postoperative pharyngeal pain. In this study, all patients underwent postoperative nebulization and oral inclusions and were discharged with significant symptom relief.

#### 4.2.1 Protection of other nerves by IONM

Patients with damage to the outer branch of the SLN tend to have decreased pitch (43). There are several anatomical variants between the outer branch of the SLN and the superior(STA) thyroid artery, and there are many ways of typing. The accepted international typing standard is the Cernea (44). The outer branch of the SLN may pass below the upper margin of the superior thyroid pole and cross the STA, which is known as Cernea 2B, with an incidence of about 5% to 48.3% (45–47), in which the outer branch of the SLN passes in close relation to the superior parathyroid gland. Treatment of an abnormally enlarged upper parathyroid gland in patients with SHPT and PHPT can lead to nerve injury if not performed correctly. Cernea 2B SLN accounted for 25.39% (82/323) of cases in this group. IONM was used to monitor the continuity of the SLN throughout the procedure without injury. As there is currently no method to study SLN injury, only the patient’s complaints of choking on water or change in tone were used and no complications occurred. Very few enlarged parathyroid glands in SHPT patients are ectopic near the cervical sheath in the lateral neck, and there is a risk of vagus nerve boot injury with PTX. The relationship between the vagus nerve, the carotid artery, and the internal jugular vein in the cervical sheath is variable (48, 49), so intraoperative attention must be paid to ectopic parathyroid glands and the course of the nerve carefully determined. Intraoperative IONM improves the identification of the vagus nerve and reduces identification time (50, 51). In our group of SHPT patients, two patients had two parathyroid glands in the unilateral cervical sheath, and one of the preoperative patients had a parathyroid gland near the vagus nerve, which could be removed without injury to the vagus nerve by using IONM.

This study has the following limitations: it is a single-center retrospective study, which may introduce selection bias and cannot exclude the effect of single-center. The sample size of patients with SHPT (53 cases) is relatively small, and all of them received IONM. Data from patients with SHPT who did not receive IONM were not obtained, nor were clinical data from thyroid cancer patients who did not use IONM collected. Therefore, no analysis was performed on the data of patients with SHPT and thyroid cancer, whether they used or did not use IONM, to investigate the protective effect of IONM on the recurrent laryngeal nerve during surgery for SHPT and thyroid cancer. In the future, large-sample, multi-center, and prospective studies with random sampling are needed to better understand the role of IONM in the surgical treatment of SHPT and thyroid cancer, and to obtain more definitive research conclusions.

## 5 Conclusion

In this retrospective study, it was found that the use of IONM in PTX of PHPT and SHPT patients can rapidly identify and localize the laryngeal nerve intraoperatively, determine the functional integrity of the nerve by electromyographic signals, analyze the mechanism of nerve injury, and reduce the risk of laryngeal nerve injury due to irregular parathyroid hyperplasia, anatomical abnormalities of the laryngeal nerve, and tissue adhesions. IONM is undoubtedly a better tool in PTX of SHPT and PHPT patients. More clinicians and patients will benefit from the comprehensive and accurate application of IONM in PTX.

## Data availability statement

The original contributions presented in the study are included in the article/supplementary material. Further inquiries can be directed to the corresponding authors.

## Ethics statement

The authors are accountable for all aspects of the work in ensuring that questions related to the accuracy or integrity of any part are appropriately investigated and resolved. The study was conducted by the Declaration of Helsinki (as revised in 2013). The study was approved by the Institutional Review Board of China-Japan Union Hospital of Jilin University (approval number: 20230630016). The patients or their legal guardians sign a detailed informed consent form before surgery.

## Author contributions

YM: Conceptualization, Data curation, Formal Analysis, Investigation, Methodology, Project administration, Software, Supervision, Validation, Visualization, Writing – original draft, Writing – review & editing. XB: Writing – original draft, Writing – review & editing. JY: Data curation, Formal Analysis, Validation, Visualization, Writing – original draft. YL: Writing – review & editing. YuZ: Writing – review & editing. GD: Writing – review & editing. YiZ: Funding acquisition, Methodology, Supervision, Writing – review & editing. HS: Funding acquisition, Investigation, Methodology, Resources, Writing – review & editing.
